# Prognostic impact of postoperative neutrophil-to-lymphocyte ratio on survival outcomes of patients treated with radical nephroureterectomy for upper urinary tract urothelial carcinoma: a single institution retrospective analysis using propensity score matching

**DOI:** 10.7150/jca.76977

**Published:** 2023-05-05

**Authors:** Hyung Suk Kim, Hui Seung Lee, Ja Hyeon Ku

**Affiliations:** 1 Department of Urology, Dongguk University Ilsan Medical Center, Dongguk University College of Medicine, Goyang-si, Gyeonggi-do, Korea; 2 Department of Biostatistics, Dongguk University College of Medicine, Goyang-si, Republic of Korea; 3 Department of Urology, Seoul National University Hospital, Seoul National University College of Medicine, Seoul, Korea

**Keywords:** Carcinoma, transitional cell, nephroureterectomy, neutrophil to lymphocyte ratio, systemic inflammatory response, survival

## Abstract

**Purpose:** To investigate the prognostic impact of postoperative neutrophil to lymphocyte ratio (NLR) on survival outcomes in upper urinary tract urothelial carcinoma (UTUC).

**Materials and methods:** Data from 397 patients with UTUC who underwent radical nephroureterectomy (RNU) without a history of neoadjuvant chemotherapy between 2002 and 2017 were retrospectively analyzed. Based on a postoperative NLR cut-off of 3, patients were divided into low NLR (<3) or high NLR (≥ 3) groups. After 2:1 propensity score matching, a Kaplan-Meier with log-rank test was used to compare survival outcomes between the two groups. Univariate and multivariate Cox proportional hazard models were used to investigate the impact of the postoperative NLR on survival outcomes.

**Results:** The matched cohort (n=176) consisted of 116 low NLR and 60 high NLR patients. The Kaplan-Meier curves showed significant differences in the 3- and 5-year overall and cancer-specific survival rates between the two groups (each *p* = 0.03). Multivariate Cox regression analysis revealed that a postoperative high NLR was an independent predictor of worse overall survival (hazard ratio [HR]:2.13; 95% confidence interval [CI]:1.18-3.85, *p* = 0.012) and cancer-specific survival (HR:2.16; 95% CI 1.11-4.21, *p* = 0.024).

**Conclusions:** Propensity score matching analysis revealed postoperative high NLR can be considered a potential inflammatory biomarker for predicting survival outcomes of UTUC patients treated with RNU.

## Introduction

Upper urinary tract urothelial carcinoma (UTUC) is a rare malignant tumor. Although it accounts for only 5-10% of all urothelial carcinomas (UCs), it generally shows aggressive clinical behaviors with a high potential for disease recurrence and eventual death 1, 2. Radical nephroureterectomy (RNU) with ipsilateral bladder cuff excision has long been the gold standard local treatment modality for high-risk, nonmetastatic UTUC 3. Despite this aggressive local modality, long-term outcomes usually remain poor because of disease recurrence and distant metastasis. In particular, muscle-invasive disease (pT2 or higher) generally shows a worse prognosis, with a 5-year cancer-specific survival (CSS) rate of < 50% for pT2/pT3 disease and < 10% for pT4 1, 4. Such poor outcomes for UTUC suggest a need for risk stratification based on prognostic factors that can predict clinical outcomes so that proper additional treatment modalities, such as systemic chemotherapy in a perioperative setting, could be selected for UTUC patients.

Several prognostic factors have been reported to be associated with UTUC. Tumor-related factors such as pathological tumor stage, tumor grade, carcinoma in situ (CIS), lymphovascular invasion (LVI), and lymph node (LN) involvement (LNI) have been established as primary determinants of postoperative prognosis in patients with UTUC 5-7. In addition to these tumor-related factors, the systemic inflammatory response (SIR) plays an important role in tumor development, progression, and metastasis. Many SIR-related biomarkers have been evaluated as potential prognostic factors for UTUC 8-10. Among these inflammatory biomarkers, the neutrophil-to-lymphocyte ratio (NLR) has been vigorously assessed as a potential predictive tool in relation to oncologic outcomes, such as postoperative intravesical recurrence and survival outcomes of patients with UTUC 11-19.

Previous studies have generally suggested that an increased NLR is associated with poor oncological outcomes 11-18. However, most studies have evaluated the prognostic value of the NLR in the preoperative setting. To the best of our knowledge, few studies have determined the prognostic value of the postoperative NLR in UTUC. Therefore, the objective of this study was to evaluate the prognostic impact of postoperative NLR on survival outcomes of patients with UTUC treated with RNU using propensity score (PS) matching methodology.

## Materials and Methods

### Selection of study population

The study design and use of patient information were approved by the Institutional Review Board of Seoul National University Hospital. A total of 458 patients who underwent surgery between 2002 and 2017 were initially identified in the UTUC database. After excluding patients who underwent ureterectomy (n = 22), had histology other than UC (n = 6), had a history of neoadjuvant chemotherapy (n = 8), had metastasis at diagnosis (n = 10), and had incomplete blood test data (n = 10), a total of 397 nonmetastatic patients with UTUC who underwent RNU without a history of neoadjuvant chemotherapy were eligible for the final analysis. The patient population included in this study partially overlapped with that of our previous studies 20, 21.

### Included variables

The following clinical parameters were included as covariates: age at surgery, sex, body mass index (BMI), previous or concomitant bladder cancer, preoperative urine cytology results, preoperative hydronephrosis, surgical approach (open versus laparoscopic), bladder cuff excision, and adjuvant chemotherapy (ACH). BMI was defined as weight in kilograms divided by the square of height converted to meters. ACH was selectively performed at the physician's discretion for patients with pT2 or higher stage disease and/or who were LN-positive within three months after surgery. Several tumor-related parameters were also considered as covariates, including pathological tumor stage, tumor grade, concomitant CIS, LVI, variant histology, tumor multifocality, tumor location, surgical margin, and LNI. All surgical specimens were read by a urological pathology expert according to the 2010 American Joint Committee on Cancer staging system and the 2004 World Health Organization grading classification. To facilitate the analysis, tumor stage was categorized into localized disease (≤ pT2) and locally advanced disease (pT3/T4). LVI was defined as the movement of cancer cells into either the blood or lymphatic vessels without affecting the underlying muscular walls. Tumor multifocality referred to the synchronous presence of two or more pathologically confirmed tumors at any location. Tumor location was defined as renal pelvic, ureteral, or both.

### NLR

To derive the NLR (defined as the absolute neutrophil count divided by the absolute lymphocyte count in peripheral blood), neutrophil and lymphocyte counts were measured using blood tests before and after surgery. Preoperative values were obtained within one month before RNU. Postoperative values were measured 3-4 months after surgery. If ACH was performed, the values measured before ACH were used. In all cases, chart review confirmed that there was no subjective or objective evidence of systemic inflammation at the time of measurement. We classified all patients into high (≥ 3) or low (< 3) NLR groups using a cutoff NLR of 3.0, based on the results of previous studies 11-13.

### Postoperative follow-up regimen

The patients were followed-up according to our hospital surveillance protocol. Examinations during postoperative follow-up included history, physical examination, blood chemistry, routine urine analysis, urine cytology, and cystoscopy. Several imaging studies, including chest radiography, abdominal-pelvic computed tomography (CT), chest CT, bone scan, and magnetic resonance imaging, were selectively conducted if clinically indicated. The primary endpoints of this study were overall survival (OS) and cancer-specific survival (CSS). The OS was defined as the period from the date of surgery to the last follow-up or death. The CSS was defined as the period from the time of surgery to the direct death from cancer. The date of death was identified by reviewing medical charts and/or the annual census of the Korea National Statistical Office. The cause of death was determined by the physicians in charge and the death certificates.

### Statistical analyses

The chi-square test or Fisher's exact test was used to compare the categorical variables. The Mann-Whitney U-test was used to compare continuous variables between the two NLR groups classified by their NLR values. Continuous variables are expressed as median and interquartile range (IQR). Categorical variables are expressed as absolute numbers with relative percentages. To minimize the risk of selection bias and data heterogeneity resulting from the retrospective design, the PS matching method was used to adjust for other covariates in each NLR group. PS was calculated using a logistic regression model of seven parameters: tumor stage, tumor grade, variant histology, surgical margin, LNI, age, and sex. After PS matching with a 2 (low NLR):1 (high NLR) ratio, a Kaplan-Meier log-rank test was used to compare survival outcomes between the two NLR groups. Univariate and multivariate Cox proportional hazard models were used to evaluate the prognostic impact of NLR on each survival outcome. All statistical analyses were performed using the SAS software (version 9.4; SAS Institute, Cary, NC, USA). All reported *p*-values were two-sided, and a *p*-value < 0.05 was considered to indicate a statistically significant difference.

## Results

### Baseline characteristics of the study cohort

Table 1 shows the baseline characteristics between the two postoperative NLR groups (high NLR and low NLR) classified based on an NLR cutoff of 3. Among the 397 patients, 63 (15.9%) showed a postoperative high NLR. After 2:1 PS matching, 176 patients (low/high NLR group:116/60 patients) were identified. Significant differences between the two groups were observed in variant histology, positive surgical margins, and LNI before PS matching, but no significant differences were observed after PS matching.

### Analyzing the impact of postoperative NLR on survival outcomes

**Figure 1:**
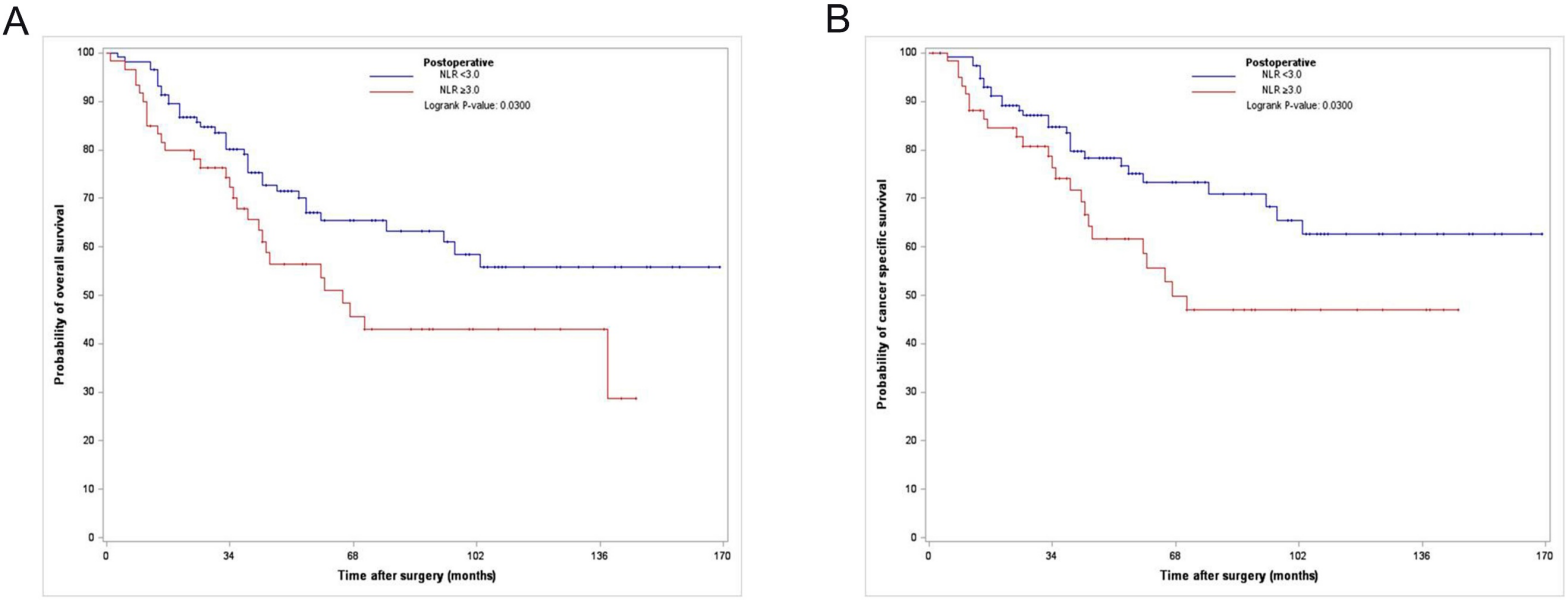
Kaplan-Meier curves for overall survival (A) and cancer specific survival (B) according to postoperative NLR in the PS matched cohort. NLR, neutrophil-to-lymphocyte ratio; PS, propensity score

Among the 176 PS-matched patients, 65 (36.9 %) died of all causes, of which 52 (29.5%) died due to UTUC (Table 1) during a median follow-up period of 41.5 months. The Kaplan-Meier analysis showed 3- and 5-year OS rates of 71.7% and 60% and 3- and 5-year CSS rates of 76.7% and 66.7%, respectively, in the postoperative high NLR group, which were significantly lower than those in the postoperative low NLR group (3- and 5-year OS rates of 89.9% and 72.4%, and 3- and 5-year CSS rates of 86.2% and 79.3%, respectively) (Fig. 1).

Univariate analysis performed in the PS-matched cohort revealed that the postoperative NLR was significantly related to OS and CSS, along with urine cytology, surgical approach, bladder cuff excision, tumor stage, tumor grade, LVI, variant histology, tumor multifocality, surgical margin, and nodal stage (Table 2). When conducting multivariate Cox regression analysis incorporating covariates, a postoperative high NLR (≥3) was an independent predictor of worse OS (hazard ratio [HR]:2.13; 95% confidence interval [CI]:1.18-3.85, *p* = 0.012) and CSS (HR:2.16; 95% CI 1.11-4.21, *p* = 0.024) (Tables 2 and 3).

## Discussion

The possible connection between inflammation and cancer was first described by Virchow in 1876, after identifying the presence of leukocytes in tumor tissues. Evidence now clearly supports that SIR plays an important role in the development, progression, metastasis, and survival of malignant cells in most cancers 22. In most solid tumors, an intrinsic inflammatory response precedes the formation of a protumorigenic microenvironment. In the tumor microenvironment, inflammation promotes processes such as angiogenesis, tumor invasion, and metastasis, which are mediated by several chemokines and cytokines secreted by various immune cells 22, 23. Based on this background, many clinical studies have been conducted on the prognostic value of inflammatory biomarkers in various solid tumors. Among these biomarkers, the NLR has been vigorously investigated as the most representative inflammatory biomarker 24-26.

The prognostic value of the NLR in UTUC has also been assessed in several studies. However, most of these studies have focused on the prognostic value of the preoperative NLR in relation to oncological outcomes, including intravesical recurrence and survival outcomes 11-18. Although the threshold to define elevated or high NLR levels was not uniform among studies (ranging from 2.5 to 3), elevated NLR showed close correlations with poor oncologic outcomes, including a higher risk of intravesical recurrence [15. 18] and unfavorable survival outcomes [11-14. 16, 17], regardless of the NLR threshold. Only a few studies have presented results on the prognostic value of the postoperative NLR in UC. In a retrospective analysis of 385 patients who underwent radical cystectomy for bladder UC, an elevated NLR (≥ 2) was significantly associated with unfavorable pathologic outcomes (such as higher disease stage (≥pT3), LVI, and LNI) and poorer OS and CSS 27. In addition, when the entire population was divided by perioperative (before and after surgery) NLR changes, the group showing pre- and postoperative elevated NLR showed poorer survival results than other groups 27. A recent study reported the prognostic significance of postoperative NLR in UTUC. In a retrospective analysis of 134 patients with UTUC who underwent RNU, a postoperative high NLR, defined as an NLR of ≥ 2.5, showed significant associations with an advanced pathologic T stage and LVI. Five-year OS (33.7% vs. 70.2%) and CSS rates (22.7% vs. 80.7%) were also significantly lower in the postoperative high-NLR group 19.

Similar to results reported in previous studies 19, 27, our study showed that a postoperative high NLR in UTUC was associated with lower survival rates after surgery. However, our study and related studies reported so far have inherent limitations in that they were all retrospective and observational rather than a prospective, randomized design. To overcome the possible selection bias and data heterogeneity resulting from the retrospective design, we used PS-matching methodology 28. In observational studies, PS matching is a commonly performed statistical analysis technique. When PS matching is performed, the distribution of covariates between two groups is balanced, and the possibility of bias is reduced 28. In this study, there was a significant difference in the distribution of variant histology, surgical margins, and LNI between the NLR groups, but there was no difference between the two groups in the variables after PS matching. Based on Cox regression analysis performed in the cohort created after PS matching, postoperative high NLR, which was defined as ≥ 3, was identified as an independent factor predicting poor OS and CSS. Unlike previous studies, the strength of our study is that it was conducted using PS matching, which in turn can contribute to the generalizability of the results derived from this study. Furthermore, our study results may help determine how to follow-up patients and whether additional treatment, such as adjuvant chemotherapy, is needed after surgery in patients with UTUC.

However, the results derived from this study should be interpreted with caution because of the following limitations. First, although the PS matching method was used, the possibility of inevitable selection bias cannot be completely ruled out because of the retrospective and non-randomized design. For instance, other lymphocyte-related biomarkers, including platelet-to-lymphocyte ratio and monocyte-to-lymphocyte ratio 17, 18 and inflammation-related hematological factors such as C-reactive protein 29 and erythrocyte sedimentation rate 14, which were assessed as significant prognostic factors for UTUC in previous studies, were not included in the present study. Second, because the surgical procedures included in this study were performed by multiple surgeons, there might be heterogeneity in the surgical process and expertise. In addition, there might be variations among surgeons with regard to the criteria for determining the implementation of ACH, type of ACH regimen, and postoperative follow-up method. These factors may have affected the postoperative survival outcomes. LN dissection, which is considered an important part of the current surgical treatment for UTUC, was not performed in a significant number (approximately 80%) of patients. Therefore, the accurate LN status may not have been evaluated. The possibility that this had an effect on the survival outcomes could not be excluded. Last, since our study data were obtained from a single institution, a validation process through a multicenter study involving a large number of patients is needed in the future to generalize the results of this study.

In conclusion, our PS-matching study results indicate that the postoperative NLR can be predictive of survival outcomes after RNU for UTUC. If the predictive role of postoperative NLR is validated through additional multicenter and prospective studies in the future, it is expected to serve as a useful tool to determine whether to follow-up and provide adjuvant treatment for patients with UTUC.

## Ethical approval of studies

The study design and use of patient information stored in the hospital database were approved by the Institutional Review Board (IRB) of the Seoul National University Hospital (approval number: H-2303-130-1414). The requirement for informed consent was waived by the IRB because this was a retrospective study with complete removal of personal identifiers, and data were analyzed anonymously. This study was conducted according to the ethical standards laid down in the 1964 Declaration of Helsinki and its later amendments.

## Author Contributions

Conception and design: Hyung Suk Kim, Ja Hyeon Ku; Data acquisition: Hyung Suk Kim, Ja Hyeon Ku; Data analysis and interpretation: Hyung Suk Kim, Ja Hyeon Ku; Drafting the manuscript: Hyung Suk Kim; Critical revision of the manuscript for scientific and factual content: Hyung Suk Kim and Ja Hyeon Ku; Statistical analysis: Hyung Suk Kim, Hui Seung Lee; Supervision: Ja Hyeon Ku.

## Figures and Tables

**Table 1:** Patients' characteristics of the study cohort; Pre and post propensity score matching data.

		Pre-propensity score matching				Post-propensity score matching		
Variables	Total (n=397)	NLR < 3 (n=334, 84.1%)	NLR ≥ 3 (n=63, 15.9%)	p-value	Total (n=176)	NLR < 3 (n=116, 65.5%)	NLR ≥ 3 (n=60, 34.5%)	p-value
***Clinical parameters***								
Age, year, median (IQR)	65.1(58.2, 71.6)	65.1(58.3, 71.6)	65.6(58.1, 70.7)	0.793	65.7(58.7, 71.4)	66 (58.7, 71.5)	65.4(58.7, 70.5)	0.935
Gender, n (%)				0.102				0.708
Male	302(76.1%)	249(74.6%)	53(84.1%)		144(81.8%)	94(81%)	50(83.3%)	
Female	95(23.9%)	85(25.4%)	10(15.9%)		32(18.2%)	22(19%)	10(16.7%)	
BMI (kg/m^2^), median (IQR)	24.3(22.9, 25.9)	24.3(22.3, 26.0)	24.4(22.1, 25.4)	0.456	24.4(22.3, 25.7)	24.3(22.5, 25.8)	24.5(22, 25.2)	0.483
Previous or concomitant bladder cancer, n (%)				0.441				0.346
Absent	320(80.6%)	267(79.9%)	53(84.1%)		146(83%)	94(81%)	52(86.7%)	
Present	77(19.4%)	67(20.1%)	10(15.9%)		30(17%)	22(19%)	8(13.3%)	
Previous urine cytology result, n (%)				0.939				0.684
Negative	194(48.9%)	164(49.1%)	39(47.6%)		89(50.6%)	59(50.9%)	30(50%)	
Positive	165(41.6%)\	139(41.6%)	26(41.3%)		71(40.3%)	49(41.4%)	23(38.3%)	
Missing/unknown	38(9.6%)	31(9.3%)	7(11.1%)		16(9.1%)	9(7.8%)	7(11.7)	
Preoperative hydronephrosis, n (%)				0.915				0.294
Absent	223(56.2%)	188(56.3%)	35(55.6%)		93(52.8%)	58(50%)	35(58.3%)	
Present	174(43.8%)	146(43.7%)	28(44.4%)		83(47.2%)	58(50%)	25(41.7%)	
Surgical approach				0.896				0.747
ORNU	280(70.5%)	236(70.7%)	44(69.8%)		123(69.9%)	82(70.7%)	41(68.3%)	
LRNU	117(29.5%)	98(29.3%)	19(30.2%)		53(30.1%)	34(29.3%)	19(31.7%)	
Bladder cuff excision				0.452				0.704
Not done	63(15.9%)	51(15.3%)	12(19.0%)		29(16.5%)	20(17.2%)	9(15%)	
Done	334(84.1%)	283(84.7%)	51(81.0%)		147(83.5%)	96(82.8%)	51(85%)	
***Pathological parameters***								
Tumor stage, n (%)				0.321				0.977
≤pT2	237(59.7%)	203(60.8%)	34(54.0%)		100(56.8%)	66(56.9%)	34(56.7%)	
pT3/T4	160(40.3%)	131(39.2%)	29(46.0%)		76(43.2%)	50(43.1%)	26(43.3%)	
Tumor grade, n (%)				0.353				0.917
Low grade	127(32.0%)	110(32.9%)	17(27.0%)		49(27.8%)	32(27.6%)	17(28.3%)	
High grade	270(68.0%)	224(67.1%)	46(73.0%)		127(72.2%)	84(72.4%)	43(71.7%)	
Concomitant CIS, n (%)				0.640				0.699
Absent	339(85.4%)	284(85.0%)	55(87.3%)		150(85.2%)	98(84.5%)	52(86.7%)	
Present	58(14.6%)	50(15.0%)	8(12.7%)		26(14.8%)	18(15.5%)	9(13.3%)	
LVI, n (%)				0.147				0.901
Absent	311(78.3%)	266(79.6%)	45(71.4%)		131(74.4%)	86(74.1%)	45(75%)	
Present	86(21.7%)	68(20.4%)	18(28.6%)		45(25.6%)	30(25.9%)	15(25%)	
Variant histology of UC, n (%)				0.003				0.813
Absent	361(90.9%)	310(92.8%)	51(81.0%)		148(84.1%)	97(83.6%)	51(85%)	
Present	36(9.1%)	24(7.2%)	12(19.0%)		28(15.9%)	19(16.4%)	9(15%)	
Tumor multifocality, n (%)				0.269				0.527
Unifocal	328(82.6%)	279(83.5%)	49(77.8%)		148(84.1%)	99(85.3%)	49(81.7%)	
Multifocal	69(17.4%)	54(16.2%)	14(22.2%)		28(15.9%)	17(14.7%)	11(18.3%)	
Tumor location, n (%)				0.064				0.605
Renal pelvis	192(48.4%)	164(49.1%)	28(44.4%)		82(46.6%)	54(46.6%)	28(46.7%)	
Ureter	148(37.3%)	128(38.3%)	20(31.7%)		65(36.9%)	45(38.8%)	20(33.3%)	
Both	57(14.4%)	42(12.6%)	15(23.8%)		29(16.5%)	17(14.7%)	12(20%)	
Surgical margin, n (%)				<0.001				0.328
Negative	376(94.7%)	323(96.7%)	53(84.1%)		158(89.8%)	106(91.4%)	52(86.7%)	
Positive	21(5.3%)	11(3.3%)	10(15.9%)		18(10.2%)	10(8.6%)	8(13.3%)	
LN status, n (%)				0.042				0.497
pNx	319(80.4%)	275(82.3%)	44(69.8%)		136(77.3%)	92(79.3%)	44(73.3%)	
pN0	63(15.9%)	49(14.7%)	14(22.2%)		30(17%)	17(14.7%)	13(21.7%)	
pN ≥1	15(3.8%)	10(3.0%)	5(7.9%)		10(5.7%)	7(6%)	3(5%)	
***Postoperative follow up parameters***								
ACH, n (%)				0.152				0.477
Not done	304(76.5%)	261(78.1%)	44(69.8%)		129(73.3%)	87(75%)	42(70%)	
Done	93(23.5%)	73(21.9%)	19(30.2%)		47(26.7%)	29(35%)	18(30%)	
Follow-up duration (mos), median (IQR)	45(24, 86)	47.5(24, 87)	36(18, 71)	0.058	41.5(24, 79)	42.5(23.5, 84)	38.5(24, 72)	0.383
OS result, n (%)				<0.001				0.024
Alive	272(68.5%)	241(72.2%)	31(49.2%)		111(63.1%)	80(69%)	31(51.7%)	
All cause death	125(31.5%)	93(27.8%)	32(50.8%)		65(36.9%)	36(31%)	29(48.3%)	
CSS result, n (%)				<0.001				0.029
Alive or other cause death	299(75.3%)	263(78.7%)	36(57.1%)		124(70.5%)	88(75.9%)	36(60%)	
Cancer-specific death	98(24.7%)	71(21.3%)	27(42.9%)		52(29.5%)	28(24.1%)	24(40%)	

**Table 2:** Univariable and multivariable Cox proportional hazard models for overall survival in the propensity score matched cohort (n=176)

	Univariable analysis		Multivariable analysis	
Variables	Unadjusted HR (95% CI)	p-value	Adjusted HR (95% CI)	p-value
***Clinical parameters***				
Age (continuous)	1.01(0.98-1.04)	0.465	1.02(0.99-1.05)	0.139
Gender (female vs. male)	0.71(0.35-1.43)	0.334	0.51(0.21-1.22)	0.130
BMI (continuous)	0.96(0.89-1.04)	0.344	1.03(0.94-1.13)	0.533
Previous or concomitant bladder cancer (present vs. absent)	1.61(0.89-2.91)	0.115	1.97(0.88-4.37)	0.097
Preoperative urine cytology (positive vs. negative)	1.79(1.06-3.01)	0.029	1.49(0.83-2.70)	0.184
Preoperative hydronephrosis (present vs. absent)	1.35(0.83-2.20)	0.230,	0.95(0.53-1.71)	0.876
Surgical approach (LRNU vs. ORNU)	2.13(1.29-3.52)	0.003	2.21(1.14-4.27)	0.019
Bladder cuff excision (done vs. not done)	0.38(0.22-0.66)	<.001	0.38(0.19-0.76)	0.006
ACH (done vs. not done)	2.34(1.41-3.90)	0.001	0.67(0.29-1.56)	0.354
Preoperative NLR (continuous)	1.02(0.92-1.15)	0.671	1.07(0.91-1.27)	0.419
Postoperative NLR (≥3 vs. <3)	1.70(1.04-2.78)	0.033	2.13(1.18-3.85)	0.012
***Pathological parameters***				
Tumor stage (pT3/4 vs. ≤pT2)	2.70(1.64-4.45)	<.001	1.50(0.71-3.16)	0.290
Tumor grade (high vs. low)	3.10(1.53-6.28)	0.002	1.94(0.79-4.74)	0.146
Concomitant CIS (present vs. absent)	0.85(0.40-1.78)	0.661	0.53(0.21-1.36)	0.188
LVI (present vs. absent)	1.88(1.12-3.15)	0.016	1.13(0.56-2.29)	0.738
Variant histology of UC (present vs. absent)	3.06(1.77-5.31)	<.001	2.60(1.20-5.64)	0.016
Tumor multifocality (multifocal vs. unifocal)	2.01(1.13-3.59)	0.018	1.95(0.70-5.39)	0.199
Tumor location (both vs. renal pelvis or ureter only)	1.56(0.88-2.74)	0.125	1.53(0.59-3.96)	0.385
Surgical margin (positive vs. negative)	4.39(2.36-8.16)	<.001	3.48(1.51-8.00)	0.003
Nodal stage (≥pN1 vs. pN0/Nx)	2.44(1.05-5.68)	0.038	2.71(0.87-8.50)	0.087

**Table 3:** Univariable and multivariable Cox proportional hazard models for cancer specific survival in the propensity score matched cohort (n=176)

	Univariable analysis		Multivariable analysis	
Variables	Unadjusted HR (95% CI)	p-value	Adjusted HR (95% CI)	p-value
***Clinical parameters***				
Age (continuous)	1.00(0.97-1.03)	0.834	1.01(0.98-1.05)	0.455
Gender (female vs. male)	0.57(0.24-1.33)	0.193	0.46(0.17-1.25)	0.129
BMI (continuous)	0.96(0.88-1.05)	0.396	1.04(0.93-1.15)	0.521
Previous or concomitant bladder cancer (present vs. absent)	1.37(0.69-2.73)	0.372	2.12(0.84-5.39)	0.114
Preoperative urine cytology (positive vs. negative)	2.42(1.32-4.43)	0.004	2.05(1.03-4.08)	0.042
Preoperative hydronephrosis (present vs. absent)	1.36(0.79-2.34)	0.271	0.95(0.49-1.85)	0.891
Surgical approach (LRNU vs. ORNU)	2.40(1.37-4.19)	0.002	1.91(0.91-4.03)	0.087
Bladder cuff excision (done vs. not done)	0.30(0.16-0.53)	<.001	0.25(0.12-0.52)	<.001
ACH (done vs. not done)	3.25(1.87-5.65)	<.001	0.99(0.38-2.58)	0.981
Preoperative NLR (continuous)	1.01(0.89-1.15)	0.875	1.03(0.84-1.27)	0.749
Postoperative NLR (≥3 vs. <3)	1.81(1.05-3.12)	0.033	2.16(1.11-4.21)	0.024
***Pathological parameters***				
Tumor stage (pT3/4 vs. ≤pT2)	2.89(1.65-5.07)	<.001	1.14(0.47-2.78)	0.777
Tumor grade (high vs. low)	4.76(1.89-12.00)	<.001	3.16(0.98-10.16)	0.054
Concomitant CIS (present vs. absent)	0.63(0.25-1.58)	0.32	0.33(0.10-1.05)	0.06
LVI (present vs. absent)	2.29(1.31-4.02)	0.004	1.07(0.50-2.33)	0.858
Variant histology of UC (present vs. absent)	4.02(2.23-7.25)	<.001	2.96(1.26-6.94)	0.013
Tumor multifocality (multifocal vs. unifocal)	1.79(0.92-3.49)	0.088	2.12(0.65-6.87)	0.212
Tumor location (both vs. renal pelvis or ureter only)	1.43(0.75-2.73)	0.277	1.40(0.47-4.16)	0.548
Surgical margin (positive vs. negative)	4.39(2.36-8.16)	<.001	2.50(0.97-6.41)	0.057
Nodal stage (≥pN1 vs. pN0/Nx)	2.56(1.01-6.47)	0.047	2.30(0.65-8.22)	0.198
